# 基于超高效液相色谱-高分辨质谱和渗透压校正样本浓度变异性的尿液代谢组学分析

**DOI:** 10.3724/SP.J.1123.2020.06018

**Published:** 2021-04-08

**Authors:** Zhian HE, Houwei LIN, Juan GUI, Weichao ZHU, Jianhua HE, Hang WANG, Lei FENG

**Affiliations:** 1.上海交通大学药学院, 上海 200240; 1. School of Pharmacy, Shanghai Jiao Tong University, Shanghai 200240, China; 2.上海交通大学分析测试中心, 上海 200240; 2. Instrumental Analysis Center, Shanghai Jiao Tong University, Shanghai 200240, China; 3.上海交通大学医学院附属新华医院小儿泌尿外科, 上海 200092; 3. Department of Pediatric Urology,Xinhua Hospital Affiliated to Shanghai Jiao Tong University School of Medicine, Shanghai 200092,China; 4.嘉兴学院附属嘉兴市妇幼保健院小儿外科, 浙江 嘉兴 314051; 4. Department of Pediatric Surgery, Jiaxing University Affiliated Women and Children Hospital, Jiaxing 314051, China; 5.宁波大学医学院附属医院小儿外科 浙江 宁波 315020; 5. Department of Pediatric Surgery, the Affiliated Hospital of Medical School of Ningbo University, Ningbo 315020, China

**Keywords:** 超高效液相色谱-高分辨质谱, 代谢组学, 渗透压, 归一化, 尿液, ultra performance liquid chromatography-high resolution mass spectrometry (UPLC-HRMS), metabolomics, osmolality, normalization, urine

## Abstract

尿液是代谢组学研究中主要关注的体液样本之一。尿液样本中的代谢物浓度受饮食、疾病等因素影响变异较大,这极大阻碍了高质量组学数据的获取和可靠生物标志物的鉴定。研究为克服尿液样本的浓度变异性,在原始数据采集前,根据样本渗透压的大小,针对性地调整进样量或者稀释样本,从而确保代谢组学分析样本的渗透压与进样量的乘积相当,再经超高效液相色谱-高分辨质谱技术(UPLC-HRMS)分析,采用总离子丰度或总有用峰面积(MSTUS)对数据集进行归一化处理。研究利用临床样本及其梯度稀释的溶液,对该方法与现有研究普遍使用的方法进行了比较,随后通过先天性肾积水患者及健康志愿者的尿液样本做了进一步的方法学验证。数据集经校正后,峰面积RSD<30%的提取峰数量增加,主成分分析结果较校正前有更高的组内聚集和组间分群效应,正交偏最小二乘判别分析的统计模型更不易过拟合。与肌酐比较,渗透压值与质谱信号间呈现了更好的线性关系。以上结果表明,数据采集前通过样本渗透压进行校正,能有效消除因样本本身代谢物浓度变化引起的组内差异,提高方法的重复性和统计模型的可靠度。以渗透压为基准的校正策略,比肌酐校正法适用范围更广,结果也更准确。研究可对后续各类来源的尿液代谢组学研究提供数据归一化的指导和参考。

## Abstract (en)

Urine is an important source of biomolecular information for metabolomic studies. However, the acquisition of high-quality metabolomic datasets or reliable biomarkers from urine is difficult owing to the large variations in the concentrations of endogenous metabolites in the biofluid, which are caused by diverse factors such as water consumption, drugs, and diseases. Thus, normalization or calibration is essential in urine metabolomics for eliminating such deviations. The urine osmolality (*Π*), which is a direct measure of the total urinary solute concentration and is not affected by circadian rhythms, diet, gender, and age, is often considered the gold standard for estimation of the urine concentration. In this study, a pre-data acquisition calibration strategy based on osmolality was investigated for its feasibility to overcome sample concentration variability. Before data acquisition, the product of the osmolality×injection volume of all samples was set to be equivalent through the uses of a customized injection volume or dilution. After ultra performance liquid chromatography-high resolution mass spectrometry (UPLC-HRMS) analysis of the sample, the raw dataset was normalized to the total ion abundance or total useful MS signals (MSTUS) to achieve further calibration. The osmolality of each urine sample was determined with a freezing-point depression osmometer. For the instrumental analysis, a Vanquish UPLC system coupled to a Q-Exactive Plus HRMS device was used for metabolite analysis and accurate mass measurement. Full-scan mass spectra were acquired in the range of *m/z* 60-900, and the MS/MS experiments were conducted in “Top5” data-dependent mode. A Waters UPLC column (100 mm×2.1 mm, 1.8 μm) was used for chromatography separation. The raw data were imported into Progenesis QI software for peak picking, alignment, deconvolution, and normalization. SIMCA-P software was used for the principal component analysis (PCA) and orthogonal partial least-squares discrimination analysis (OPLS-DA). This strategy was first applied to sequentially diluted urine samples, where three frequently used normalization methods were compared. In the identical injection volume experiment, the points were scattered and showed relevant distribution according to the dilution multiple in the plot of PCA scores. There was little improvement after normalization to either the total ion abundance or MSTUS. In the customized injection volume experiment, the urine samples derived from the same source showed ideal clustering. With total ion abundance and MSTUS normalization, the dataset was further improved in the PCA model fitting and prediction. As a result, there were more peaks with a peak area RSD of <30%, which indicated better parallelism. The diluted urine solutions had higher Spearman’s coefficient values with their sample source than those without calibration, which suggested less intra-group differences. The strategy was further validated using data from a metabolomic study of children with congenital hydronephrosis and healthy controls. As a concentration estimator, osmolality showed better linear correlation with the mass signal and was less influenced by physiological or pathological factors, thus obtaining broader application and more accurate results than creatinine. The concentration variability was effectively eliminated after customized dilution calibration and showed a more obvious clustering effect in the PCA score plot. The OPLS-DA-based statistical model used to identify discriminate metabolites was improved, with less chance of overfitting. In conclusion, the calibration strategy based on osmolality combined with total ion abundance or MSTUS normalization significantly overcame the problem of urine concentration variability, eliminated intra-group differences, and possessed better parallelism, thus giving better clustering effects in PCA or OPLS-DA and higher reliability of the statistical model. The results of this study provide guidance and a reference for future metabolomic studies on urine.

代谢组学(metabolomics)是通过对某一生物或细胞内所有相对分子质量较低(<1000)的代谢物质进行定性和定量分析,进而将内外因素作用下产生的代谢物质的动态变化与生理病理相关联的学科^[1]^。作为系统生物学的最下游,代谢组学反映了生物体受到各种内外环境扰动后的实际状态和不同个体之间的表型差异^[2]^。超高效液相色谱-高分辨质谱技术(UPLC-HRMS)具有灵敏度高、分析通量大、覆盖范围广的优势,是代谢组学采用的主要技术之一^[3]^。代谢组学在生物医学领域具有广泛的应用,例如寻找早期诊断疾病的关键指征、评估手术预后的生物标志物和开发新的药物等^[4,5]^。

尿液在临床上易大量获取,并且与血液相比,受蛋白质、脂质的干扰小且可避免人体的侵入性伤害,是临床代谢组学长期关注的体液样本^[6,7]^。尿液中的内源性代谢物浓度会因饮水、进食及其他生物因素而改变,跨度可达15倍,造成个体间非疾病因素的差异^[8]^。因此,针对尿液样本的代谢组学研究需要进行一定的归一化,以消除这一影响。

尿液代谢组学的归一化处理通常分为数据采集前归一化(以下简称“校正”)和数据采集后归一化(下简称“归一化”)两个维度^[9]^。现有研究中,校正可通过一定的浓度估计参数进行固相萃取(SPE)^[10]^、调整进样量^[11]^或稀释^[12]^等前处理过程来实现。归一化主要有肌酐值或肌酐峰面积归一化、总离子丰度(total ion abundance)、总有用峰面积(MSTUS)归一化及24 h尿液总量归一化^[9,13]^。

正常机体每天经尿液排泄的肌酐总量是恒定的,因此肌酐是尿液代谢组学研究中最常被使用的校正或归一化参数^[14,15]^。然而,肌酐的排泄仍可能受到性别、年龄、体重指数(BMI)、药物以及肾损伤等因素的影响,进而限制了这一方法的适用范围^[12,16,17]^。渗透压(osmolality, *Π*)的大小可以反映代谢物总量,并且受生理、环境因素的影响小,其测定可独立完成,冰点法测定快速方便,所需样本量少^[18,19]^。目前,渗透压常常仅作为数据采集后的归一化参数^[9,20]^,基于渗透压的数据采集前校正方法十分少见,有部分报道^[10]^采用渗透压校正结合固相萃取实现,前处理较为复杂,不利于重复操作,并且缺乏临床样本的评价和验证。

因此,本研究以先天性肾积水患者尿液为研究对象,建立了基于渗透压的校正方法。利用单变量和多变量统计分析,从方法的重复性、组内及组间差异、统计模型的拟合预测能力等角度对校正方法同已有研究做了比较和验证,旨在为后续的尿液代谢组学研究提供指导和参考。

## 1 实验部分

### 1.1 仪器、试剂与材料

Vanquish UPLC超高效液相色谱系统、Q Exactive plus四极杆-静电场轨道阱质谱仪(美国Thermo Fisher公司); OM-819.C冰点渗透压仪(中国雅森公司); TGL-16台式高速冷冻离心机(湖南湘仪实验仪器有限公司); ACQUITY UPLC HSS T3色谱柱(100 mm×2.1 mm, 1.8 μm,美国Waters公司)。

乙腈、甲醇(质谱级)均购自美国Thermo Fisher公司;甲酸(色谱级)购自德国CNW试剂公司;肌酐(东京化成工业株式会社);实验用水采用Milli-Q超纯水系统(美国Millipore公司)制备。

尿液样本来自2019年嘉兴学院附属嘉兴市妇幼保健院小儿外科确诊的10例先天性肾积水患者(疾病组,U)和同期性别、年龄匹配的13例健康志愿者(对照组,HC),收集后置于-80 ℃冰箱保存。本研究经嘉兴市妇幼保健院伦理委员会审定(编号:2019(伦)-69),合理可行。

### 1.2 渗透压的测定

使用纯水和标准溶液对冰点渗透压仪进行0、300及900 mOsm 3点校准,校准完成后即可开始测样。取100 μL样品,置于1.5 mL离心管中,固定样品管位置,调整高度直至探针末端完全浸没在溶液中。将样品管推至下方冷阱,仪器自动开始检测。待示数稳定后读数,弃去样品管,无尘纸擦净探针,测试下一样品。每连续测定10个样本,需测定1次纯水作0点参比,超过±3 mOsm需重新校准。

### 1.3 尿液样本的制备与校正

尿液样本从-80 ℃冰箱中移至室温下解冻,涡旋1 min。每个样本取等量混合,制备质控(QC)样本。QC样本随其他样本一起进行前处理,它反映了样本的平均水平。在进行UPLC-HRMS分析时,先连续进样10次QC样本平衡仪器,每隔10个样品插入1个QC样本,序列最后重复进样3次QC样本,用于监控仪器的灵敏度和稳定性。基于渗透压的校正,关键在于保证样本进样体积(*V*, μL)与渗透压值的乘积处于同一水平。因此校正过程可通过调整*V*或*Π*两种方式实现。

1.3.1 调整进样体积

取尿液样本500 μL,加入等体积甲醇,涡旋1 min,混合均匀,于-20 ℃静置2 h,低温离心(4 ℃, 12000 r/min)20 min,取上清液置于进样瓶中待测。假定所有样本中渗透压的最低值为*Π*_Lowest_,其余样本的渗透压值为*Π*_Sample_,则稀释倍数*F*_Volume_=*Π*_Sample_/*Π*_Lowest_。在进行仪器分析时,假定渗透压最低的样本进样量为*n*(μL),则其他样本的*V*为*n*/*F*_Volume_ (μL)。

1.3.2 稀释样本

根据实际样本的渗透压大小,确定稀释后适宜的样本渗透压*Π*_Reference_,则稀释倍数*F*_Dilution_=*Π*_Sample_/*Π*_Reference_。取样本*m* μL,加入超纯水(*F*_Dilution_-1)×*m* μL、甲醇*m*×*F*_Dilution_ μL,涡旋1 min,混合均匀,于-20 ℃静置2 h。静置完成后,低温离心(4 ℃, 12000 r/min)20 min,取上清置于进样瓶中待测。在进行仪器分析时,等体积进样。

### 1.4 分析条件

1.4.1 色谱条件

色谱柱:Waters HSS T3色谱柱(100 mm×2.1 mm, 1.8 μm);柱温:40 ℃;流动相:(A)0.1%(v/v)甲酸水溶液和(B)0.1%(v/v)甲酸乙腈溶液;流速:0.4 mL/min,样品室温度:8 ℃。线性梯度洗脱程序:0~1.0 min, 1%B; 1.0~7.5 min, 1%B~20%B; 7.5~9.5 min, 20%B~30%B; 9.5~13.5 min, 30%B~35%B; 13.5~15.5 min, 35%B~70%B; 15.5~16.0 min, 70%B~100%B; 16.0~18.0 min, 100%B; 18.0~18.1 min, 100%B~1%B; 18.1~20.0 min, 1%B。等体积进样实验,进样量设为5 μL;校正进样体积实验,进样量按照1.3.1节计算调整。

1.4.2 质谱条件

离子源为加热电喷雾电离(HESI)源;喷雾电压为3.2 kV;毛细管温度为320 ℃;鞘气流速为55 arb;辅助气流速15 arb;不设反吹气;加热温度350 ℃。在正离子扫描模式下获得代谢物指纹图谱;质谱采集模式为轮廓(profile)模式;全扫描范围为*m/z* 60~900;全扫分辨率为70000;自动增益控制(AGC)设为1×10^6^个离子;离子注入时间为100 ms。MS/MS在Top5模式下进行数据依赖型采集( DDA);分辨率为17500;最大进样时间为50 ms;隔离窗口*m/z* 1.5;碰撞能为(30±15)eV。样品在分析时随机进样,以避免仪器波动造成的影响。

### 1.5 数据处理

1.5.1 数据预处理

将原始数据(. raw格式)导入Progenesis QI软件中进行峰提取、峰对齐、去卷积等数据预处理。导入数据时,过滤阈值设定为1.0,参比选择QC样本,峰提取的加合形式选择M+H、M+2H、M+Na和M+NH_4_。对导出的数据矩阵进行过滤,最终保留的提取峰应同时满足:1)“80%规则”,在一类组别中80%的样本响应不为0; 2)QC样本中峰面积变异系数(CV)<30%。

1.5.2 归一化

不同的归一化法通过在Progenesis QI软件的“Normalization method”项下选择不同的参数实现。具体设置如下,1)肌酐峰面积归一化:外标确证肌酐*m/z*为114.0667(±5 ppm(10^-6^)),保留时间(0.71±0.05) min。以每个样本中肌酐的提取峰面积做归一化;2)总离子丰度归一化:选择“Total ion abundance”,以样本中所有离子丰度的总和进行归一化;3)MSTUS归一化:选择所有样品中共有的离子,以其峰面积总和进行归一化。

1.5.3 多变量统计分析

经预处理后的数据矩阵导入SIMCA-P 14.0,经Par标准化处理后,进行主成分分析(PCA)和有监督的正交偏最小二乘法判别分析(OPLS-DA)。对OPLS-DA模型做200次置换检验,考察是否存在过拟合。

## 2 结果与讨论

### 2.1 校正方法的评价

将3个健康志愿者的尿液样本分别梯度稀释2、4、8和12倍,以模拟实际情况下尿液的浓度变化。所有样本测定渗透压,并进行前处理,分别直接等体积进样和校正体积进样后,进行UPLC-HRMS分析。校正进样体积的具体信息见表1,经过校正后,所有样本的渗透压与进样体积的乘积处于同一水平。

**表 1:** 样本的渗透压及进样体积

Dilution multiple	Sample A				Sample B				Sample C			
	*Π*/mOsm	*V*/μL	*Π*×*V*/(mOsm·μL)		*Π*/mOsm	*V*/μL	*Π*×*V*/(mOsm·μL)		*Π*/mOsm		*V*/μL	*Π*×*V*/(mOsm·μL)
1	593	0.5	296.5		644	0.5	322.0		455		0.7	318.5
2	304	1.0	304.0		326	1.0	326.0		232		1.3	301.6
4	152	2.0	304.0		163	1.9	309.7		115		2.7	310.5
8	74	4.2	310.8		82	3.8	311.6		38		8.2	311.6
12	51	6.1	311.1		53	5.9	312.7		31		10.0	310.0

2.1.1 样本及其稀释溶液的主成分分析

校正前后所得的数据集经不同归一化处理后,进行主成分分析,所得PCA得分图见图1,相同颜色和形状的点表示来源于同一样本。未经校正时,样本点散落并与稀释倍数呈现一定的规律,仅通过归一化无法改善同源样本的聚集情况。经过校正后,样本及其稀释溶液间的浓度差异被有效消除,呈现了良好的组内聚集和组间分群效应。肌酐峰面积作为尿液代谢组学常用的归一化参数,在校正前后均未得到理想结果,有研究表明,这可能与肌酐的排泄易受其他生理病理因素影响有关^[15]^。

**图 1:**
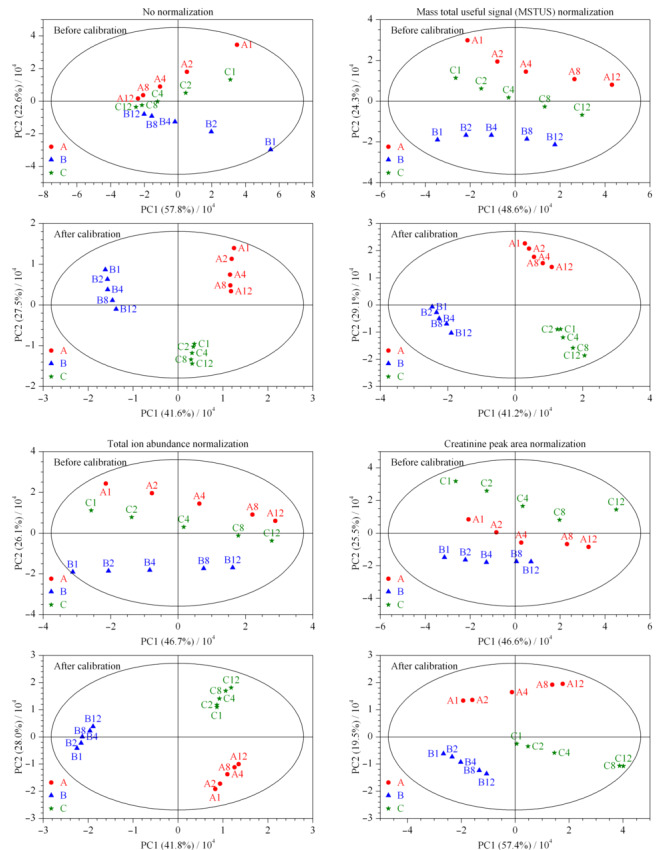
校正前、后尿液样本及其稀释溶液的PCA得分图(PC个数=4)

2.1.2 校正及归一化对重复性的影响

原始的数据矩阵经预处理共得到7930个有效提取峰。经不同归一化处理后,分别统计3个样本及其稀释溶液中峰面积RSD值<30%的峰数量,结果见图2。根据配对*t*检验结果,确定方法间是否存在显著性差异。峰面积RSD值<30%的峰数量在校正以后显著增加,经总离子丰度归一化或MSTUS归一化可进一步提高。因此,渗透压校正结合总离子丰度归一化或MSTUS归一化可有效改善方法的重复性。

**图 2:**
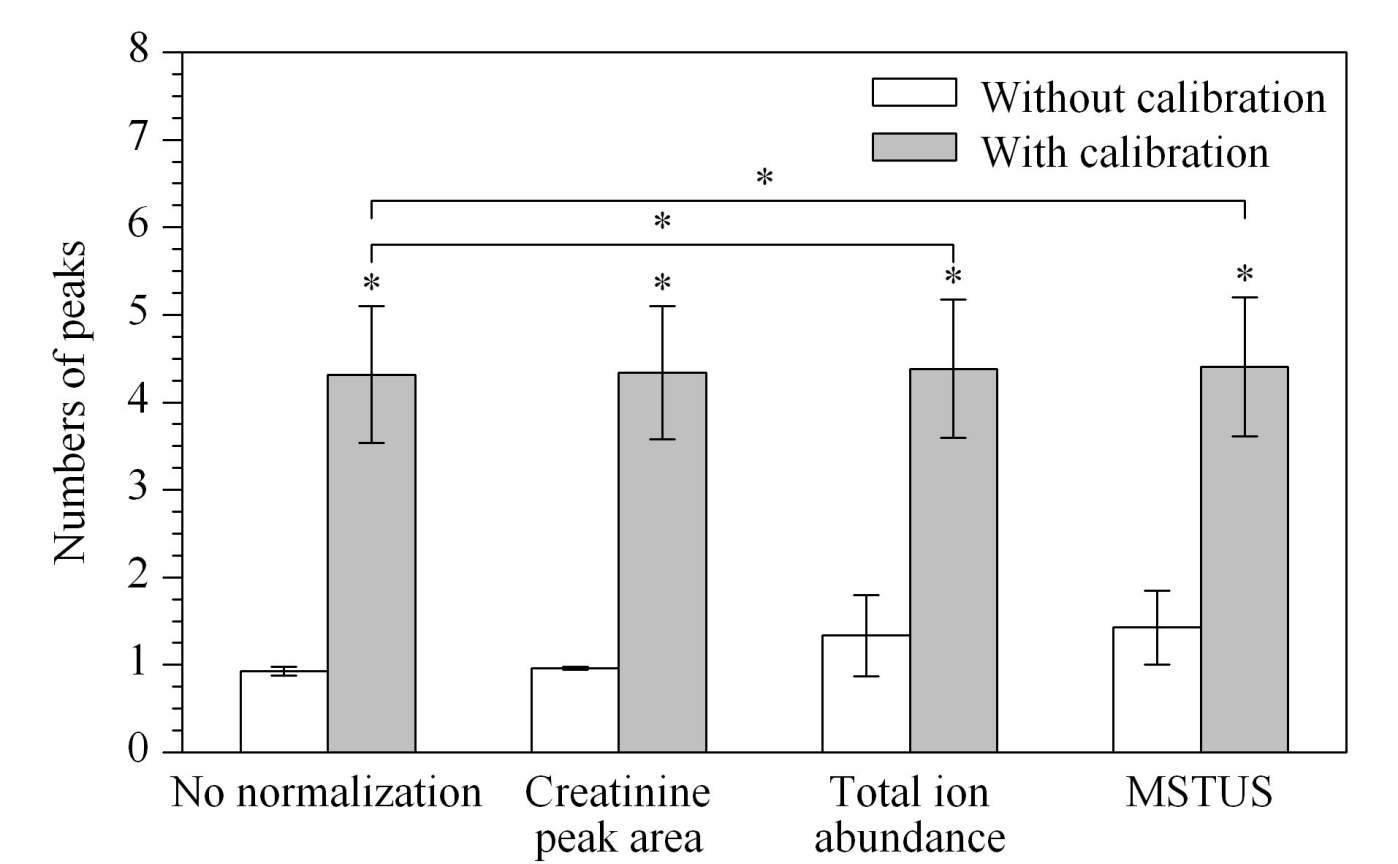
峰面积RSD<30%的提取峰数量(*n*=3)

2.1.3 同源样本的相关性分析

利用斯皮尔曼秩和相关系数(Spearman coefficient)评价样本间代谢谱的相似程度,系数越接近于1,说明样本间的相似程度越高^[21,22]^。稀释溶液与其原样本的Spearman相关系数随稀释倍数的变化如图3所示,所有相关性经检验后*p*值均小于0.05。与不经校正比较,经校正后的相关系数受稀释倍数影响更小,始终具有更好的相关性,并维持在较高水平(>0.8)。说明该校正方法可一定程度上减少尿液本身浓度带来的组内差异,使得样本更接近于原样本的真实状态。

**图 3:**
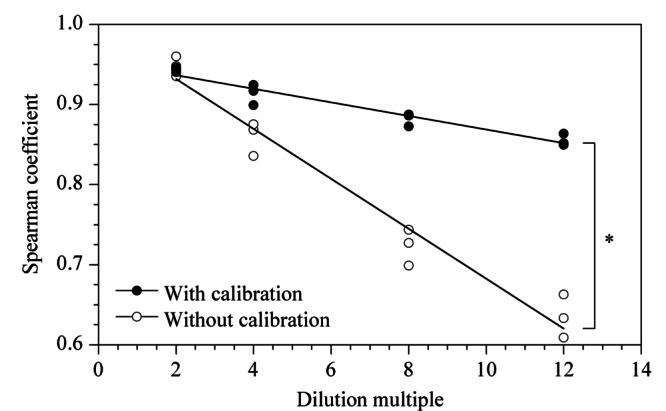
样品及其梯度稀释溶液的Spearman相关系数

### 2.2 校正方法的代谢组学验证

以上实验利用尿液样本及其梯度稀释溶液对方法进行了初步评价。然而,对于稀释的样本进行校正过程,近似于对同一样本重复进样。本研究以10例先天性肾积水患者作为疾病组, 10位健康志愿者作为对照组,做进一步的方法学验证。尿液样本分别经等体积进样和稀释至同一渗透压水平后等体积进样两次,然后进行UPLC-HRMS分析。

2.2.1 两种浓度估计参数的比较

肌酐值和渗透压值都可被用于估计尿液代谢物的浓度^[12]^。对照组与疾病组样本的肌酐峰面积和渗透压分布如图4所示,两组的渗透压没有显著性差别,但疾病组的肌酐峰面积显著低于对照组。有研究表明,肾功能的变化会影响尿肌酐的浓度,在某些情况下,利用肌酐来归一化尿液代谢组学数据,结果并不可靠^[15]^。

**图 4:**
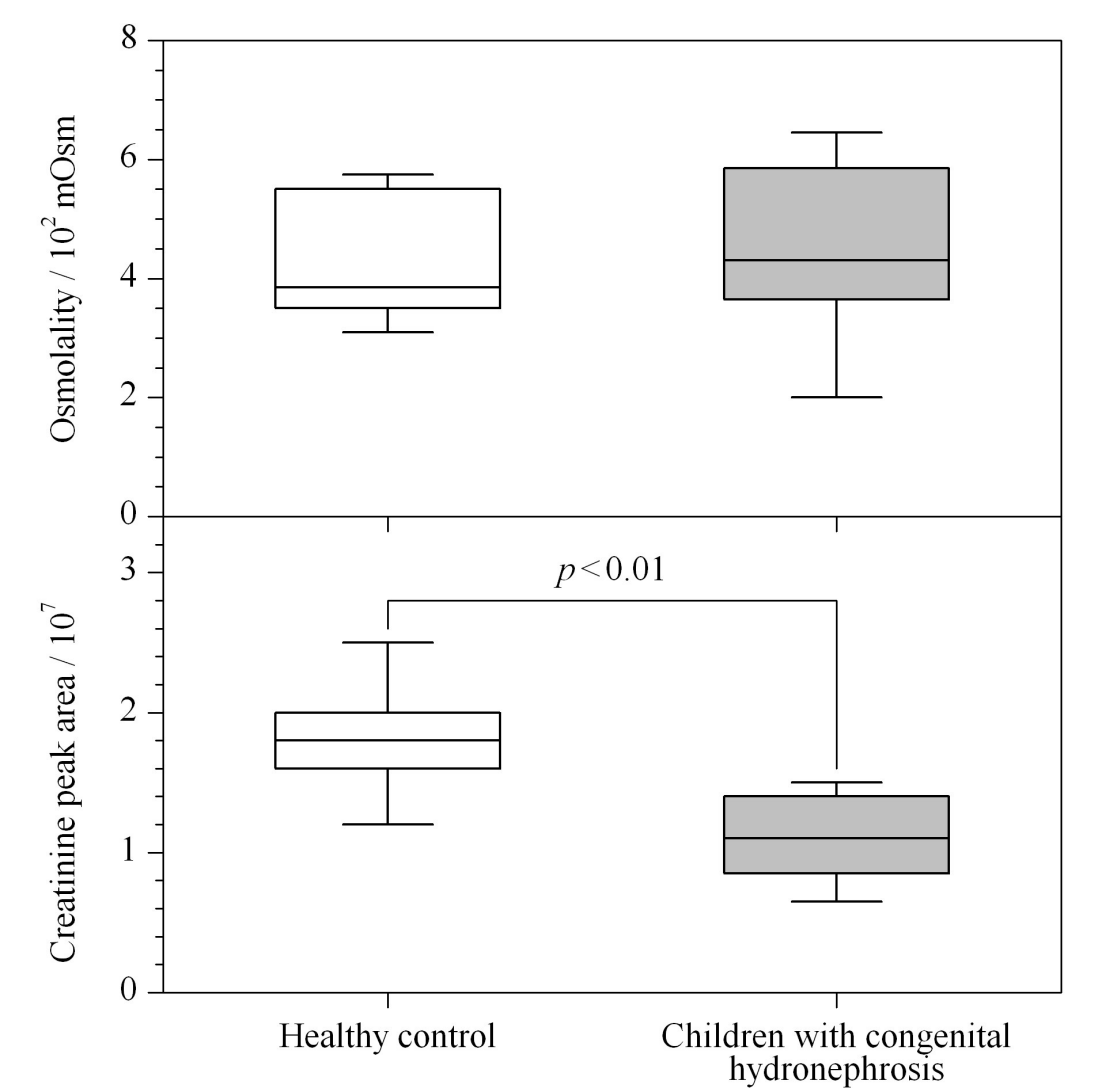
样本渗透压值和肌酐峰面积的箱型图(*n*=10)

此外,进行校正过程的关键是浓度估计参数与质谱信号间始终良好的线性相关。利用皮尔逊相关系数评价两组数据间的线性关系,相关系数越接近1,两组数据间的线性相关性越强^[23,24]^。肌酐峰面积与总峰面积仅在健康志愿者样本中线性相关,在患者和整体中无显著相关性(*p*>0.05)。说明当患者肾脏发生病变影响到肌酐的排泄时,继续使用肌酐作为浓度估计参数可能会产生较大偏差。而渗透压与总峰面积间的相关系数始终大于0.8(*p*<0.01),呈现了良好的线性。还有研究提到,社会人口学和医学条件对尿渗透压的影响小于对尿肌酐的影响^[25]^。因此,使用渗透压估计尿液代谢物的总浓度,结果更为准确,且不易受到疾病和外界条件的干扰。

2.2.2 主成分分析

用PCA比较两次代谢组学实验的结果。红色圆圈表示QC样本(Q),聚集在原点附近,表明方法的重复性良好。绿色三角代表HC,蓝色方块代表U,颜色越深表明其对应样本的渗透压越高。如图5所示,未经校正时,样本呈现了与渗透压大小相关的分布,经校正后,样本点的分布趋势与渗透压无明显相关,并有更明显的组内聚集和组间分群效应。

**图 5:**
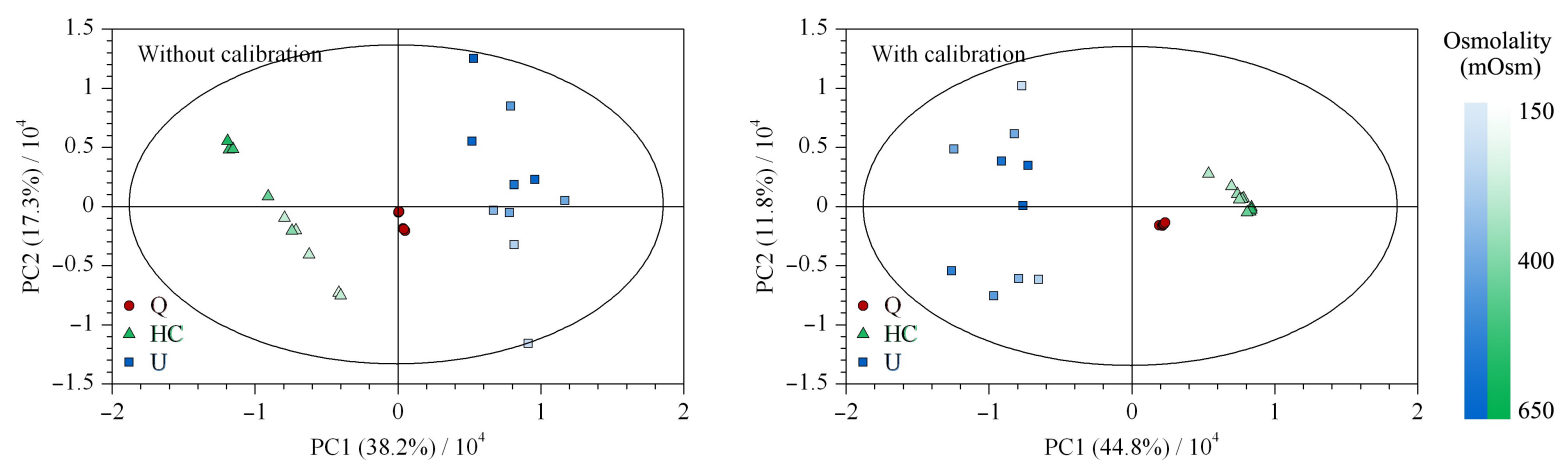
健康志愿者和先天性肾积水患者尿液样本的PCA得分图(PC个数=5)

2.2.3 OPLS-DA

OPLS-DA可以去除与分类变量无关的组内差异,使得分类信息主要集中在一个主成分(*Y*)中,在代谢组学中常被用于筛选组别间的差异代谢物,因此模型的可靠性十分重要^[26,27]^。

统计模型的*R*^2^*Y*表示模型所能解释*Y*变量信息的百分比,*Q*^2^通过交叉验证得出,分别用以评价模型的拟合和预测能力。经过校正以后,健康对照组和疾病组之间在OPLS-DA模型上有了更加明显的区分,并且*R*^2^*Y*和*Q*^2^值更加接近于1,表明其拟合和预测能力得到了提升(见图6)。利用置换检验可得到一系列OPLS-DA模型变量解释率*R*^2^的计算值,从而拟合出一条直线。直线在*Y*轴的截距越大,模型存在过拟合的可能性越高^[28]^。增加主成分的个数,对模型进行200次置换检验。经校正处理后的OPLS-DA模型,*R*^2^截距始终更小,更不易出现过拟合的情况。

**图 6:**
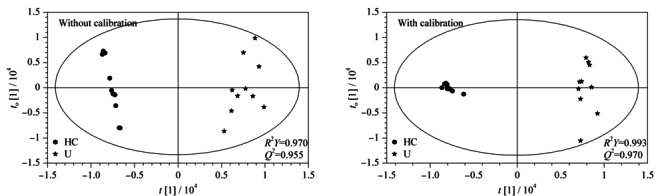
健康志愿者和肾积水患者尿液样本的OPLS-DA得分图(PC个数=2)

## 3 结论

本研究提出了一种在UPLC-HRMS数据采集前基于渗透压校正,结合总离子丰度或MSTUS归一化的策略,可克服尿液样本本身的浓度变异性。经过临床样本的验证表明,该方法有效提高了代谢组学方法的重复性,消除了因尿液本身代谢物浓度变化引起的组内差异,在PCA上拥有更明显的组内聚集和组间分群效应,并且提高了OPLS-DA模型的可靠度,更不易出现过拟合。此外,以渗透压为基准的校正方法,受疾病因素的影响小,比肌酐校正法的适用范围更广,结果更加可靠。本研究可对后续各类来源的尿液代谢组学研究提供归一化的参考和指导。
